# Twelfth International Symposium on Recent Advances in Environmental Health Research

**DOI:** 10.3390/ijerph13050470

**Published:** 2016-05-04

**Authors:** Paul B. Tchounwou

**Affiliations:** National Institutes of Health RCMI—Center for Environmental Health, College of Science, Engineering and Technology, Jackson State University, 1400 Lynch Street, Box 18750, Jackson, MS 39217, USA; paul.b.tchounwou@jsums.edu; Tel.: +1-601-979-0777; Fax: +1-601-979-0570

**Keywords:** international symposium, environmental science, biomedical science, public health

## Abstract

During the past century, environmental hazards have become a major concern, not only to public health professionals, but also to the society at large because of their tremendous health, socio-cultural and economic impacts. Various anthropogenic or natural factors have been implicated in the alteration of ecosystem integrity, as well as in the development of a wide variety of acute and/or chronic diseases in humans. It has also been demonstrated that many environmental agents, acting either independently or in combination with other toxins, may induce a wide range of adverse health outcomes. Understanding the role played by the environment in the etiology of human diseases is critical to designing cost-effective control/prevention measures. This special issue of *International Journal of Environmental Research and Public Health* includes the proceedings of the *Twelfth International Symposium on Recent Advances in Environmental Health Research*. The Symposium provided an excellent opportunity to discuss the scientific advances in biomedical, environmental, and public health research that addresses global environmental health issues.

This special issue of *International Journal of Environmental Research and Public Health* is dedicated to the publication of selected papers presented at the Twelfth International Symposium on Recent Advances in Environmental Health Research. The Symposium was organized by Jackson State University (JSU) from 13–16 September 2015 at the Marriott Hotel in Jackson, Mississippi. It was built upon the overwhelming success of eleven previous symposia hosted by JSU.

The Twelfth International Symposium on Recent Advances in Environmental Health Research served as a platform to discuss and share knowledge on the latest developments and scientific advances in biomedical, environmental health and public health. This scientific conference continued to attract biomedical, environmental health and public health scientists and engineers who are at the forefront of research and innovation in the exciting field of environmental health, and who are dedicated to providing a scientific basis for informed decision-making regarding the cost-effective control and/or prevention of environmental hazards and environmentally-induced diseases [1,2,3,4,5]. It also offered excellent opportunities for networking, sharing ideas, and sustaining and/or establishing new research collaborations and partnerships [1,2,3,4,5]. 

The previous eleven symposia (2004 to 2014) provided a solid foundation for the implementation of important activities of Twelfth International Symposium on Recent Advances in Environmental Health Research. As in the part, productive discussions were made on many important topics that included: Emerging Topics in Computational Biology and Environmental Modeling; Environmental Toxicology and Health Risk Assessment; Health Disparities and Environmental Security; Medical Geology and human Health; Nanoscience, Nanotechnology and Nanotoxicology, Natural Resources Damage Assessment and Management; and New Frontiers in Environmental Health Research [1,2,3,4,5].

The Twelfth International Symposium attracted two hundred and ninety six participants coming from twenty different countries in five continents. A total of one hundred sixty three scientific presentations were made across the disciplines of environmental health, biomedical and clinical sciences, and public health. Original contributions were solicited on relevant Symposium topics, and submitted manuscripts were subject to a rigorous peer-review process conducted according to the guidelines and high standards for publication in *International Journal of Environmental Research and Public Health* (*IJERPH*) [6]. *IJERPH* has a commendable 2014-impact factor of 2.063, and is considered as one of the premier journals advancing research that addresses critical issues related to environmental quality and public health. This high quality journal is covered by leading indexing services including PubMed (Medline) and the Science Citation Index Expanded (Web of Science), EMBASE and Scopus (SciVerse). Full-text articles are also available through PubMed Central [6].

I wish to extend special thanks to Dr. Edmond E. Creppy (Professor and Director of Toxicology Laboratory at the University of Bordeaux, Bordeaux, France), and Dr. John A. McLachlan (Professor in the Departments of Pharmacology, Ecology, and Evolutionary Biology, Tulane University Health Sciences Center, New Orleans, LA, USA), for serving as distinguished speakers for the Biomedical Sciences and Health Information Lecture Series that was held in conjunction with the Symposium. Dr. Creppy spoke on “What a Scientist Can Do to Address Food Security and Food Safety”, and Dr. McLachlan presented on “Alteration of Differentiation of Adult Human Stem Cells by Endocrine Disrupting Environmental Chemicals”. Other plenary presentations and keynote addresses were made by prominent biomedical and environmental health scientists and engineers with research expertise in cancer, diabetes, HIV/AIDS, infectious and parasitic diseases, cardiovascular diseases, neurodegenerative diseases, gene-environment interactions, nanoscience and nanomedicine, emerging technologies, health disparities and other environmentally-related illnesses. These important health issues were associated with the symposium topics [1,2,3,4,5]. 

I would like to commend the authors for their excellent contributions and for providing the scientific basis for advancing research and making informed decisions for environmental stewardship and public health protection. Special thanks are extended to all our peer-reviewers who helped to critique the research and improve the scientific quality. 

Special thanks are extended Honorable Phil Bryant (Governor of the State of Mississippi), Honorable Tony Yarber (Mayor of the City of Jackson), Dr. Carolyn W. Meyers (JSU President), Dr. Loretta Moore (JSU Vice President for Research and Federal Relations), and Dr. Richard Alo (JSU Dean of College of Science, Engineering and Technology) for presenting greetings to symposium participants. I would also like to thank all my colleagues and staff who worked very hard to make the symposium successful.

The next Symposium will be held at the Jackson Marriott Hotel (Jackson, MS, USA), from 11–14 September 2016. I strongly encourage previous Symposium participants, as well as biomedical and environmental health scientists and engineers who are dedicated to addressing global environmental and public health challenges, to joint us at the next conference in September 2016 as we celebrate the 13th anniversary of this grand event.
